# Which gait training intervention can most effectively improve gait ability in patients with cerebral palsy? A systematic review and network meta-analysis

**DOI:** 10.3389/fneur.2022.1005485

**Published:** 2023-01-10

**Authors:** Guoping Qian, Xiaoye Cai, Kai Xu, Hao Tian, Qiao Meng, Zbigniew Ossowski, Jinghong Liang

**Affiliations:** ^1^Department of Sport, Gdansk University of Physical Education and Sport, Gdańsk, Poland; ^2^Department of General Education, Shanghai Normal University Tianhua College, Shanghai, China; ^3^Department of Art and Sports, Huanghe Science and Technology University, Zhengzhou, Henan, China; ^4^Department of Physical Education and Humanities, Nanjing Sport Institute, Nanjing, China; ^5^Department of Maternal and Child Health, School of Public Health, Medical College of Sun Yat-sen University, Guangzhou, China

**Keywords:** gait, walking speed, rehabilitation, motor skills disorders, randomized controlled trials

## Abstract

**Background:**

A vital objective to treat people with cerebral palsy (CP) is to increase gait velocity and improve gross motor function. This study aimed to evaluate the relative effectiveness of gait training interventions for persons with CP.

**Methods:**

Studies published up to October 26, 2022 were searched from four electronic databases [including Medline (*via* PubMed), Web of Science, Embase and Cochrane]. Studies with randomized controlled trials (RCTs), people with CP, comparisons of different gait training interventions and outcomes of gait velocity and gross motor function measures (GMFM) were included in this study. The quality of the literature was evaluated using the risk of bias tool in the Cochrane Handbook, the extracted data were analyzed through network meta-analysis (NMA) using Stata16.0 and RevMan5.4 software.

**Results:**

Twenty RCTs with a total of 516 individuals with CP were included in accordance with the criteria of this study. The results of the NMA analysis indicated that both external cues treadmill training (ECTT) [mean difference (MD) = 0.10, 95% confidence interval CI (0.04, 0.17), *P* < 0.05] and partial body weight supported treadmill training (BWSTT) [MD = 0.12, 95% CI (0.01, 0.23), *P* < 0.05] had better gait velocity than over ground gait training (OGT), BWSTT [MD = 0.09, 95%CI(0.01,0.18), *P* < 0.05] had a better gait velocity than robot-assisted gait training (RAGT), BWSTT [MD = 0.09, 95% CI (0.06, 0.13) *P* < 0.05] had a better gait velocity than treadmill training (TT), and BWSTT [MD = 0.14, 95% CI (0.07, 0.21), *P* < 0.05] had a better gait velocity than conventional physical therapy (CON). The SUCRA ranking indicated that BWSTT optimally improved the gait velocity, and the other followed an order of BWSTT (91.7%) > ECTT (80.9%) > RAGT (46.2%) > TT (44%) > OGT (21.6%) > CON (11.1%). In terms of GMFM, for dimension D (GMFM-D), there was no statistical difference between each comparison; for dimension E (GMFM-E), RAGT [MD = 10.45, 95% CI (2.51, 18.40), *P* < 0.05] was significantly more effective than CON. Both SUCRA ranking results showed that RAGT improved GMFM-D/E optimally, with rankings of RAGT (69.7%) > TT (69.3%) > BWSTT (67.7%) > OGT (24%) > CON (20.3%), and RAGT (86.1%) > BWSTT (68.2%) > TT (58%) > CON (20.1%) > OGT (17.6%) respectively.

**Conclusion:**

This study suggested that BWSTT was optimal in increasing the gait velocity and RAGT was optimal in optimizing GMFM in persons with CP. Impacted by the limitations of the number and quality of studies, randomized controlled trials with larger sample sizes, multiple centers, and high quality should be conducted to validate the above conclusion. Further studies will be required to focus on the total duration of the intervention, duration and frequency of sessions, and intensity that are optimal for the promotion of gait ability in this population.

**Systematic review registration:**

https://doi.org/10.37766/inplasy2022.10.0108, identifier: INPLASY2022100108.

## Introduction

Cerebral palsy (CP) refers to a group of disorders attributed to non-progressive brain dysfunction in the developing fetus or infant, and it is characterized by central motor and postural dysplasia (1, 2). It has been found as the most common cause of physical disability in children, and its prevalence has still been ranging from 2 and 3.5 per thousand for the past 40 years (2). In accordance with International Classification of Diseases (ICD-11) of the WHO, The code of CP was L1-8D2, and the types consisted of spastic (8D20), dyskinesia (8D21), ataxia (8D22), Worster-Drought syndrome (8D23), as well as other specific CP (8D2Y) and unspecific CP (8D2Z) (3). persons with CP are usually classified by the Gross Motor Function Classification System (GMFCS) according to the severity of activity limitation (4). This is a useful tool for determining the level of motor ability, guiding treatment decisions and assessing motor development. CP is a vital factor leading to children's physical dysfunction, self-care barrier and social participation barrier, and it poses a heavy economic burden to children and their families in medical treatment, rehabilitation and education (5).

People with CP are commonly limited in the performance of activities of daily living (ADL; e.g., outdoor walking, stair climbing, as well as self-care activities). Since the limitations of movement and self-care are often correlated with lower limb injuries, a vital goal of treatment in individuals with CP is to improve gait ability and gross motor function (6). Existing studies have suggested that the current main strategies of treating CP consist of drug therapy, surgery and rehabilitation (7–10). However, drug therapy and surgery have certain side effects (e.g., delirium and dizziness) (11, 12). Scientific evidence has suggested that functional therapy characterized by significant similarity in motor skills is effective in improving motor function in children suffering from CP (13, 14). Rehabilitation approaches offers several treatment options (e.g., walking on the floor, treadmill walking, as well as robot-assisted gait training) (15–17). This type of training based on the intensity and repetition of exercise contributes to the recovery and improvement of posture and motor function of patients with neurological diseases (18, 19). Recent published systematic reviews and meta-analyses have suggested that gait training is highly effective in improving gait abilities (e.g., gait endurance and stride length) in people with CP and have revealed that gait training is most effective in increasing the gait velocity in persons with CP (11, 20, 21). Walking combines information from vestibular, visual and proprioceptive sources to identify the body spatially, while engaging in postural control. Gait training stimulates proprioception and thus facilitates the activation of the fulcrum and balance responses required to maintain and adjust posture. The increased gait velocity in individuals with CP may be due to muscle strengthening and activation of proprioceptive information (22, 23).

Previously published Cochrane Reviews draw a conclusion that the use of mechanically assisted walking training and treadmill training interventions may increase the walking speed, enhance the gross function, and accelerate the acquisition of motor skills (24, 25). Although existing studies have generally shown a benefit of gait training on gait capacity in persons with CP, due to the lack of high quality RCTs and the long age of publication, there has been insufficient evidence to recommend the use of different types of gait training in the clinical setting. Furthermore, studies combining the different types of gait training and grading their relative effectiveness on the walking ability of CP patients are unknown. Therefore, in the face of various interventions, conventional meta-analysis limited by pair comparison can no longer provide effective method support for the selection of optimal interventions. Network meta-analysis (NMA) is developed from conventional meta-analysis, i.e., from the comparison of two standard treatment factors to the comparison of multiple treatment factors simultaneously. Its main function is to comprehensively evaluate and rank multiple interventions at the same time (26). To help physiotherapists and clinicians make clinical decisions, they may wish to know, on average, “the optimal treatment”, so a comprehensive and up-to-date systematic review should be conducted on the relative effectiveness of gait ability intervention programmes in patients with CP. Using NMA, this study aimed to evaluate and compare the effects of different approaches of gait training on gait ability in CP patients. The specific aim of this study was to verify the relative effectiveness of different gait interventions on the gait ability of people with CP.

## Materials and methods

### Search strategy and study selection

A systematic review was conducted in accordance with the Preferred Reporting Items for Systematic Reviews and Meta-Analyses (PRISMA) Extension Statement for systematic reviews incorporating network meta-analyses (27). Either some or all data generated or analyzed in this study are included in this published article or in the data repositories listed in References. The study protocol has been registered retrospectively in the INPLASY (Registration number: INPLASY2022100108). The searching was independently conducted by two authors (GQ and XC). Medline (*via* PubMed), Embase, Web of Science (WOS) and Cochrane databases from inception to 31 December 2021 were searched extensively using the following key search terms, including (cerebral palsy OR CP) AND (walk^*^ OR gait^*^ OR feedback OR treadmill training) AND random^*^ AND control^*^ AND (walk^*^ ability OR gait^*^ ability OR gross motor function OR GMFM). All analyses were based on previously published studies and did not require ethical approval or patient consent. All searches were limited to RCTs in humans, and no language limits were set. Moreover, the reference lists cited in relevant systematic reviews and included trials were screened. In addition, the two authors each manually searched the proceedings of major international conferences, systematic reviews, meta-analysis and gray literature to recursively search potential studies to prevent missing relevant studies for which only abstracts are available. Furthermore, all initial search results were screened by two blinded investigators independently. Duplicates and articles not satisfying the selection criteria based on title and abstract were removed using EndnoteX9 (Thompson ISI Research Soft, Philadelphia, PA, USA). Next, full-text articles of all remaining studies were independently screened by two blinded investigators (HT and QM) for inclusion. Disagreements regarding inclusion were resolved through discussion or arbitration by a professor (ZO).

### Inclusion/exclusion criteria

Eligibility criteria were defined in accordance with the PICOS framework (28). Inclusion criteria were as follows: (I) Patients diagnosed with cerebral palsy (CP; spastic, dyskinesia, ataxia, Worster-Drought syndrome, other specific CP, and unspecific CP); (II) Interventions consisted of any functional gait training; (III) Comparators involved another class of gait training or a conventional physical therapy; (IV) The outcomes of interest were gait-related measures; (V) RCTs published without year and language restriction (e.g., cross-over and cluster randomized trials) were selected. Exclusion criteria were as follows: (I) If most enrolled patients are undergoing other treatments at the same time; (II) Non-randomized controlled such as case-control study, cohort study, qualitative research, full-text but unpublished, study protocol. We excluded the literature whose full text is not obtained through various channels and the data in the study cannot be used and literature that could not be utilized, such as literature with repeated publication, low quality and too little reported information.

### Data extraction and quality evaluation

The data were independently extracted by two blinded investigators (GQ and XC) from the included RCTs using a standardized data extraction form. The following data parameters were extracted from the respective RCT, which comprised name of the primary author, population, number of participants in each study, characteristics of the intervention (e.g., schedule, frequency and/or duration of intervention), age (mean or median), gender, outcome type (gait-related outcome measures including: gait velocity, gross motor function) at baseline and at last observation to obtain their change scores. All the included RCTs were coded, and any discrepancy in the extracted data was resolved through discussion between pairs of authors and where appropriate, the divergences were determined objectively by an experienced expert from our team.

The methodological quality of included RCTs was evaluated using the Cochrane Collaboration's Risk of Bias (ROB) approach (29). Two investigators (GQ and XC) independently performed the ROB evaluation on the included RCTs. Cochrane Manual 5.1.0 criteria mainly evaluated study bias through the following aspects: (I) Randomization method; (II)Allocation hiding; (III) Blind the participants and the study implementers; (IV) Blind method was applied to the results evaluators; (V) The integrity of the result data; (VI) Selective reporting of research results; (VII) Other bias. In accordance with the above criteria, the included literature was judged as “low risk”, “high risk” and “unclear”. The above evaluation was carried out independently by two researchers at first, and the controversial literature was decided whether to be included or not by a professor (ZO). ROB was evaluated in Review Manager (Version 5.4).

### Outcome measures and interventions

For the RCTs, all reported outcome indicators relating to gross motor function and gait ability were evaluated, with the primary outcome of (I) gait velocity, and the secondary outcome of (II) Gross Motor Function Measures (GMFM). The GMFM fell into functional dimensions relating specifically to standing ability (dimension D) and walking ability (dimension E). The above have been extensively used, valid and reliable measures of walking ability in CP patients (30).

In addition, to define the intervention type nodes of the network, two authors (GQ and HT), PhD students in physical culture, classified the gait interventions after reaching a consensus process. Specific gait interventions partial body weight supported treadmill training (BWSTT), robot-assisted gait training (RAGT), treadmill training (TT), external cues treadmill training (ECTT), over ground gait training (OGT) and conventional physical therapy (CON), were assigned into six different nodes since this systematic evaluation aimed to compare different gait interventions, instead of studying the effects of intervention dose or intensity. It is noteworthy that we analyzed OGT and CON separately as different interventions, due to in this study CON stands for treatments such as static stretching of lower limbs' muscles and resistance training, etc. not include any form over ground gait training. In contrast, OGT stands for studies that expressively distinguished and provided gait rehabilitation as a traditional over-ground gait training approach.

### Data synthesis and analysis

The advantage of the NMA over conventional paired meta-analyses is that a combination of direct and indirect evidence can be employed to increase the reliability of the evidence when there is no evidence to directly compare differences in the effectiveness of different interventions (31).

First, being the most important supposition in NMA, network transitivity evaluation would have a direct influence in this study for further analysis (32). Consequently, to ensure that the interventions produced relevant comparisons, which make effective provision for inferences, we compared the methodological features of all included studies, such as patients and experiment designs, to assess the transitivity supposition. The features of the participants, such as the intervention course of gait training which modified the effect, were additionally inspected. The geometry of each evidence network was summarized with a network plot for each outcome. Nodes and edges were weighted relative to the number of available treatment structures and comparisons. To be specific, edges represent head-to-head comparisons between treatments, the thickness of which is proportional to the number of direct treatment comparisons. Nodes represent specific comparators whose size is proportional to the number of direct comparisons containing that treatment node. As indicated by treatment nodes with no edge connections, there have been rare studies directly comparing the above treatments. Subsequently, all nodes (comparators) a priori were identified. Intervention outcomes were then ranked using the area under the cumulative ranking curve (SUCRA), where SUCRA serves as an indicator of the likelihood of the intervention, with closer to 100% indicating better effectiveness of the intervention. Sub-component stratified analysis was conducted to explore the interventions with the best efficacy based on SUCRA values (33). Prior to analysis, the authors further independently checked the completeness and accuracy of the extracted data when analyzing the database. In addition, comparative adjusted funnel plots were generated to detect the presence of any major types of bias (e.g., publication bias, selective reporting, or other bias). Lastly, this NMA examined the absolute differences between direct and indirect estimates in the respective closed loop by inconsistency factors (IF), i.e., inconsistency tests were performed on closed loops formed by studies with direct and indirect evidence to determine the inconsistency factor in the respective closed loop. This identified inconsistencies in the network loops, with IF values close to 0 and 95% CIs, with 0 suggesting a low probability of inconsistency (34). All analyses were conducted using Stata/SE 16.0 (Stata Corporation, Lakeway, Texas, USA) for NMA. Random effects models were employed for all indicators, requiring the use of the “MVMETA” and “Network” packages.

## Results

### Search results and study characteristics

**Figure 1:**
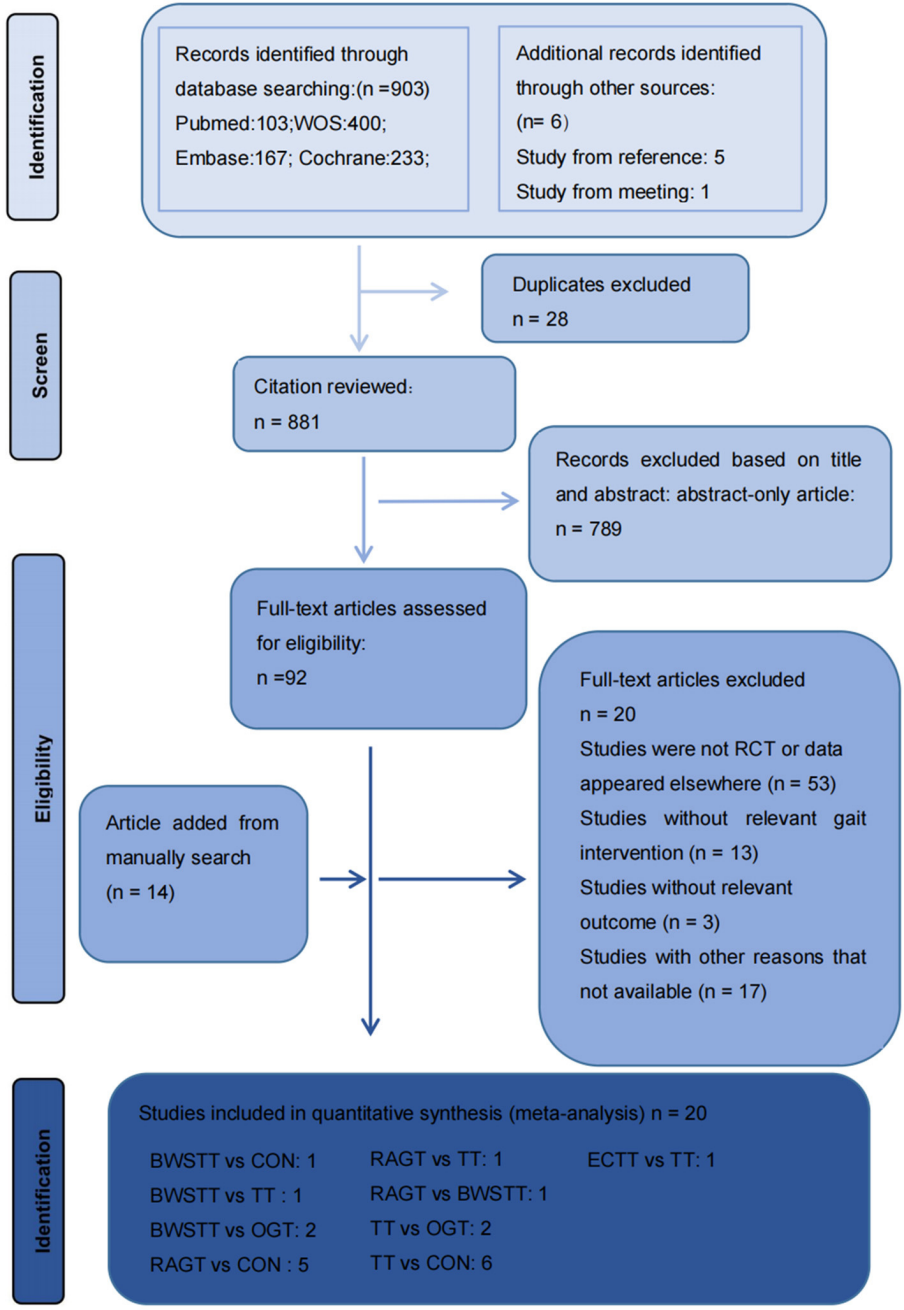
Literature review flowchart. BWSTT, partial body weight supported treadmill training. C, conventional physical therapy; ECTT, external cues treadmill training; OGT, over ground gait training; RAGT, robot-assisted gait training; TT, treadmill training; WOS, Web of Science.

Figure 1 depicts the processing of the literature selection. In the initial search, 903 relevant papers were yielded, 28 duplicates were removed, 789 were removed by reading the titles and abstracts, and 66 were excluded after the full text was red. 20 studies (33–52) satisfied the inclusion criteria with an overall sample of 516 patients (54% intervention treated; 46% control treated), with the age range from 6 to 25 years, including 216 males (42%) and 191 females (37%). Five articles were not concerned with the gender of 109 participants (0.21%). A total of 15 (75%) studies (35–37, 40–45, 48–53) have examined the gait velocity as the outcome, and 9 (45%) studies (35, 37, 40, 42, 43, 46, 47, 52, 54) have examined GMFM as the outcome. RCTs were published between 2007 and 2021. The maximum intervention time ranged from 4 weeks to 12 weeks. Participants exercised with a frequency of three times per week in 7 studies, 2 times per week in six studies, five times per week in six studies, in one study 2–5 times per week. Most RCTs were from Europe (*n* = 7), followed by Africa (*n* = 4), the Americas (*n* = 3), Australia (*n* = 3), and Asia (*n* = 3). Participants recruited by RCTs primarily come from educational and rehabilitation health facilities, hospitals and specialist schools. Table 1 lists the key characteristic of participants and interventions across the 20 included studies.

**Table 1:** Characteristics of the included studies.

**Study**	**Sample size**	**Gender**	**Mean age (SD)**	**Population**	**Intervention**	**Session duration**	**Session frequency**	**Intervention length**	**Region**
Cho et al. (35)	E: 9 C: 9	NR	E: 10.2 (3.4) C: 9.4 (3.8)	Spastic CP GMFCS level: E:I = 3/II = 1/III = 5 C:I = 3/II = 2/III = 4	E: ECTT C: TT	E: 30 min C: 30 min	3 times/week	8 weeks	Korea
Emara et al. (36)	E: 10 C: 10	E: 7F 3M C: 6F 4M	E: 6.6 (0.7) C: 6.9 (0.6)	Spastic Diplegic CP GMFCS level: E:III = 10 C:III = 10	E: BWSTT C: TT	E: 30 min C: 30 min	3 times/week	12 weeks	Egypt
Willoughby et al. (37)	E: 12 C: 14	E: 6F 6M C: 9F 5M	E: 10.35 (3.14) C: 11.24 (4.17)	CP GMFCS level: E:III = 5/IV = 7 C: III = 3/IV = 11	E: BWSTT C: OGT	E: 30 min C: 30 min	2 times/week	9 weeks	Australia
Tingting et al. (38)	E: 17 C: 17	E: 9F 8M C: 7F 10M	E: 9.82 (2.68) C: 8.27 (2.74)	Spastic CP GMFCS level: NR	E: RAGT C: CON	E: 60 min C: 60 min	5 times/week	8 weeks	China
Klobucká et al. (39)	E: 21 C: 26	E: 10F 11M C: 10F 16M	E: 18.3 (3.84) C: 23.4 (5.33)	Bilateral Spastic CP GMFCS level: E:I = 1/II = 3/III = 9/IV = 8 C:II = 4/III = 12/IV = 10	E: RAGT C: CON	E: 55 min C: 55 min	3–5times/week	4-6 weeks	Slovakia
Bahrami et al. (40)	E: 15 C: 14	E: 7F 8M C: 6F 9M	E: 25.9 (7.7) C: 25.1 (4.3)	Spastic CP GMFCS level: E:I = 7/II = 2/III = 6 C:I = 8/II = 2/III = 5	E: TT C: CON	E: 40 min C: 40 min	2 times/week	8 weeks	Iran
Ameer et al. (41)	E: 10 C: 10	NR	E: 6.2 (1.35) C: 6.2 (1.07)	Spastic Diplegic CP GMFCS level: NR	E: TT C: CON	E: 60 min C: 40 min	3 times/week	8 weeks	Egypt
Druzbicki et al. (42)	E: 26 C: 9	NR	E: 10.1 (2.2) C: 11 (2.3)	Spastic Diplegic CP GMFCS level: E:II = 15/III = 11 C:II = 8/III = 1	E: RAGT C: CON	E: 40 min C: 40 min	5 times/week	4 weeks	Poland
Swe et al. (43)	E: 15 C: 15	E:5F 10M C:5F 10M	E: 13.03 (3.56) C: 13.37 (3.32)	CP GMFCS level: E:II = 10/III = 5 C:II = 8/III = 7	E: BWSTT C: OGT	E: 30 min C: 30 min	2 times/week	8 weeks	Australia
Wallard et al. (44)	E: 14 C: 16	E: 6F 8M C: 9F 7M	E: 8.3 (1.2) C: 9.6 (1.7)	CP GMFCS level: NR	E: RAGT C: CON	E: 40 min C: 40 min	5 times/week	4 weeks	France
Smania et al. (45)	E: 9 C: 9	E: 5F 4M C: 3F 6M	E: 13.88 (2.83) C: 12.79 (3.08)	diplegic or tetraplegic CP GMFCS level: E:I = 3/II = 2/IV = 4 C:I = 3/III = 3/IV = 3	E: RAGT C: CON	E: 40 min C: 40 min	2 times/week	5 weeks	Italy
Chrysagis et al. (46)	E: 11 C: 11	E: 5F 6M C: 4F 7M	E: 15.9 (1.97) C: 16.09 (1.51)	Spastic CP GMFCS level: E:I = 3/II = 4/III = 4 C:I = 2/II = 5/III = 4	E: TT C: CON	E: 30 min C: 45 min	3 times/week	12 weeks	Greece
Wu et al. (47)	E: 11 C: 12	E: 5F 6M C: 4F 8M	E: 11.3 (3.8) C: 10.5 (2.6)	CP GMFCS level: E:I = 1/II = 6/III = 3/IV = 1 C:I = 2/II = 3/III = 5/IV = 2	E: RAGT C: TT	E: 30-40 min C: 30-40 min	3 times/week	6 weeks	America
Grecco et al. (48)	E: 16 C: 18	E: 10F 6M C: 8F 9M	E: 6.8 (2.6) C: 6.0 (1.5)	CP GMFCS level: E:I = 5/II = 8/III = 3 C:I = 8/II = 7/III = 2	E: TT C: OGT	E: 30 min C: 30 min	2 times/week	4 weeks	Brazil
Johnston et al. (49)	E: 13 C: 13	NR	9.6 (2.2)	spastic diplegic, triplegic or quadriplegic CPGMFCS level: NR	E: TT C: CON	E: 60 min C: 60 min	5 times/week	12 weeks	America
Gharib et al. (50)	E: 15 C: 15	E: 5F 10M C: 9F 6M	E: 11.87 (1.06) C: 11.23 (1.11)	Hemiparetic CP GMFCS level: NR	E: TT C: CON	E: 30 min C: 30 min	3 times/week	13 weeks	Egypt
Hamed et al. (51)	E: 15 C: 15	13 F 17 M	E: 7.03 (0.76) C: 7.07 (0.82)	Hemiparetic CP GMFCS level: NR	E: TT C: OGT	E: 60 min C: 60 min	5 times/week	12 weeks	Egypt
Hösl et al. (52)	E: 5 C: 5	NR	12 (4)	CP GMFCS level: NR	E: TT C: CON	E: 60 min C: 60 min	3 times/week	9 weeks	Germany
Aras et al. (53)	E: 10 C: 10	E: 4F 6M C: 4F 6M	9.3(2.3)	CP GMFCS level: E:I = 9/II = 1 C:I = 3/II = 7	E:RAGT C:BWSTT	E: 45 min C: 45 min	5 times/week	4 weeks	Turkey
Dodd et al. (54)	E: 7 C: 7	E: 5F 2M C: 5F 2M	E: 8.5 (2.6) C: 9.5 (2.9)	Bilateral Spastic CP GMFCS level: E:III = 2/IV = 5 C:III = 2/IV = 5	E:BWSTT C:CON	E: 30 min C: 30 min	2 times/week	6 weeks	Australia

BWSTT, partial body weight supported treadmill training; C, controlled group; CON, conventional physical therapy; CP, cerebral palsy; ECTT, external cues treadmill training; E, experimental group; F, female; GMFCS, Gross Motor Function Classification System; M, male; NR, not reported; OGT, over ground gait training; RAGT, robot-assisted treadmill training; TT, treadmill training.

### Quality evaluation

**Figure 2:**
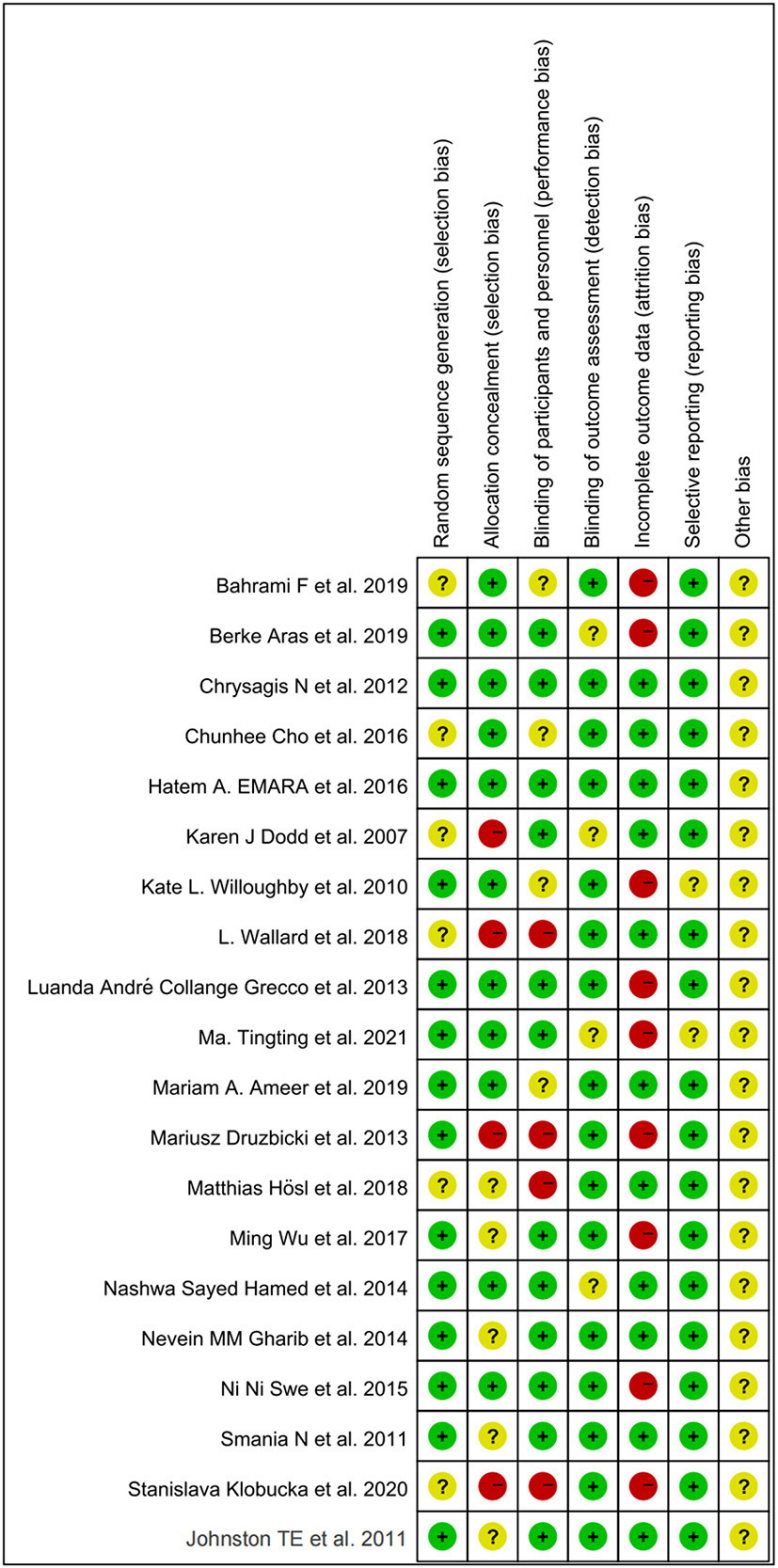
Individual projects of included articles that produced a risk of bias.

All included studies were RCTs. Random sequence generation was adequately reported in 14 studies, and six studies did not adequately report on how randomization is performed. A total of four studies did not mention distributive hiding, eleven studies were double-blind, and the rest were alluded to blindness. A total of 11 studies showed good data integrity. Only two studies did not mention selective outcome reporting. Other biases were uncertain. Individual and overall study-level quality are plotted in Figure 2 and Supplementary Figure S1, respectively.

### NMA results

#### Primary outcome

**Figure 3:**
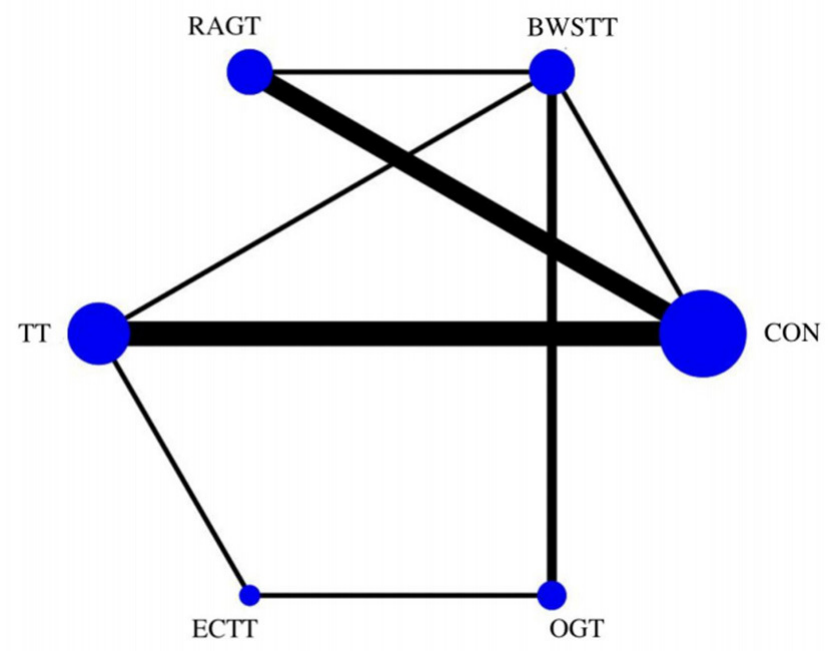
Evidence network of gait velocity analysis. BWSTT, partial body weight supported treadmill training; C, conventional physical therapy; ECTT, external cues treadmill training; OGT, over ground gait training; RAGT, robot-assisted gait training; TT, treadmill training.

Gait velocity was reported in 15 studies involving six interventions: CON, BWSTT, RAGT, TT, ECTT and OGT with a total of 378 patients, resulting in eight direct comparison studies, with three closed loops formed between interventions. The results indicated that the maximum number of studies comparing TT with CON was five, and the sample size of studies comparing TT with CON was the largest (107 patients). Figure 3 illustrates the evidence network. Six interventions were directly compared in a NMA of the included studies, and the results of the NMA suggested that gait velocity is significantly higher after the BWSTT [MD = 0.09, 95% CI (0.01, 0.18) *P* < 0.05] intervention compared with the RAGT, and significantly lower after the TT intervention compared with the BWSTT [MD = 0.09, 95%CI (0.06, 0.13) *P* < 0.05]. Gait velocity after OGT intervention was lower than that after BWSTT [MD = 0.12, 95% CI (0.01, 0.23), *P* < 0.05] and ECTT [MD = 0.10, 95% CI (0.04, 0.17), *P* < 0.05]. BWSTT [MD = 0.14, 95% CI (0.07, 0.21), *P* < 0.05] achieved a higher gait speed than CON after the intervention. The differences between the remaining groups did not achieve statistical significance (*P* > 0.05) in a two-way comparison, as listed in Table 2.

**Table 2:**
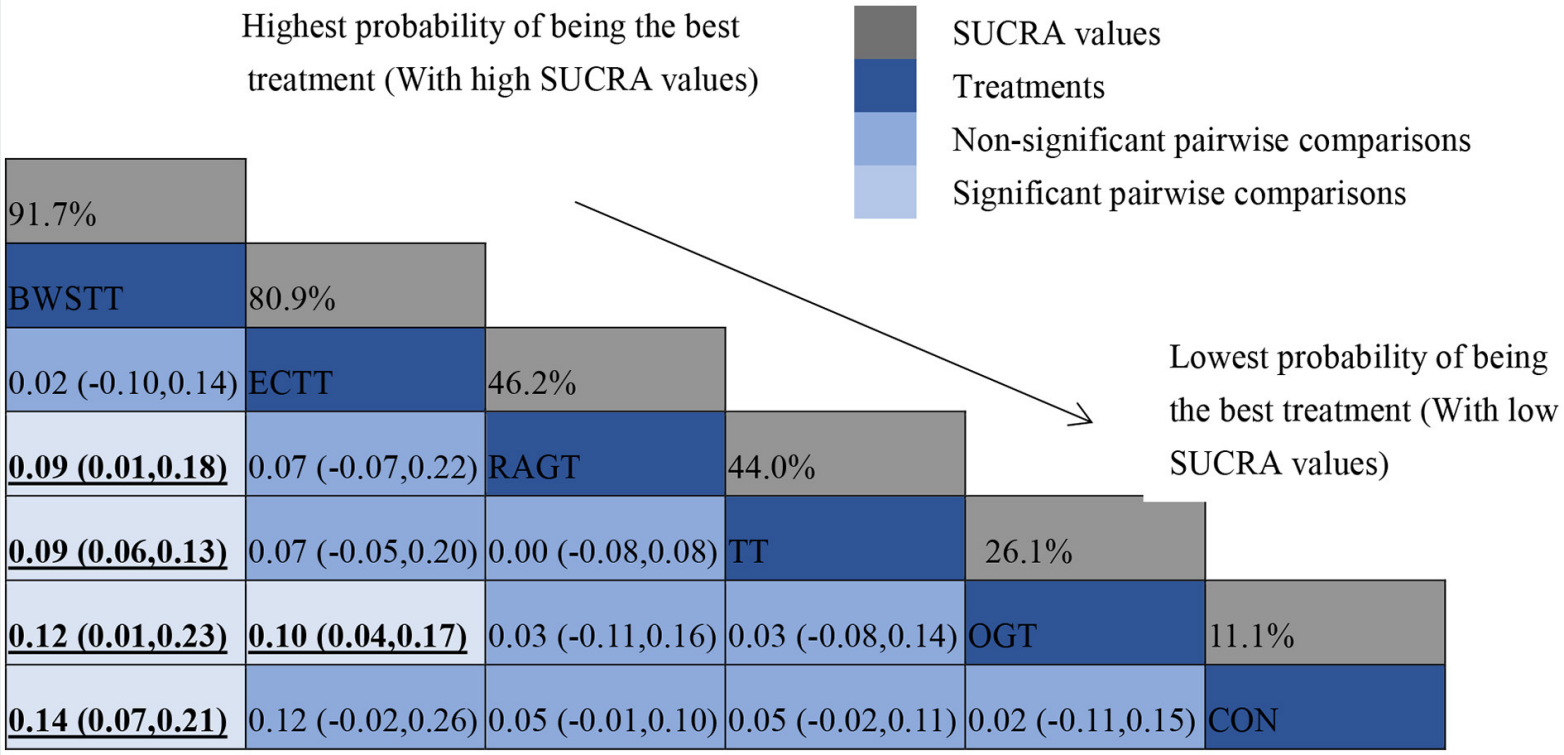
Relative effect sizes of efficacy at post-intervention according to network meta-analysis.

Interventions are orders in the rank of their chance of being the best intervention. Numbers in gray boxes are SUCRA (the surface under the cumulative ranking curve) values, which represented the rank of the treatment. Significant pairwise comparisons are highlighted in bold and underlined (MD with 95% CI). BWSTT, partial body weight supported treadmill training; C, conventional therapy; ECTT, external cues treadmill training; OGT, over ground gait training; RAGT, robot-assisted gait training; TT, treadmill training.

Intervention effects were ranked in accordance with cumulative SUCRA. The BWSTT optimal intervention was the most likely. The cumulative probability ranking was BWSTT (91.7%) > ECTT (80.9%) > RAGT (46.2%) > TT (44%) > OGT (21.6%) > CON (11.1%), as presented in Supplementary Figure S2 Comparative-corrected funnel plots show that all studies were largely distributed on both sides of the midline, with a roughly symmetrical left-right distribution, suggesting no strong publication bias, as presented in Supplementary Figure S3. Loop inconsistency analysis was conducted for the outcome indicators, suggesting that loop is less likely to exist inconsistency, as presented in Supplementary Figure S4.

#### Secondary outcome

**Figure 4:**
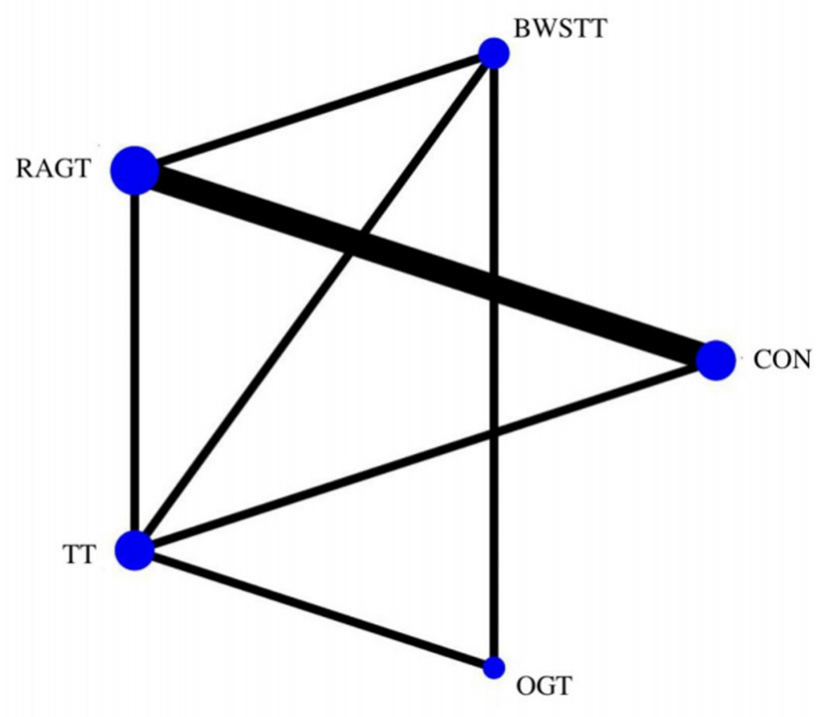
Evidence network of GMFM-D analysis. BWSTT, partial body weight supported treadmill training; C, conventional physical therapy; OGT, over ground gait training; RAGT, robot-assisted gait training; IT, treadmill training.

**Figure 5:**
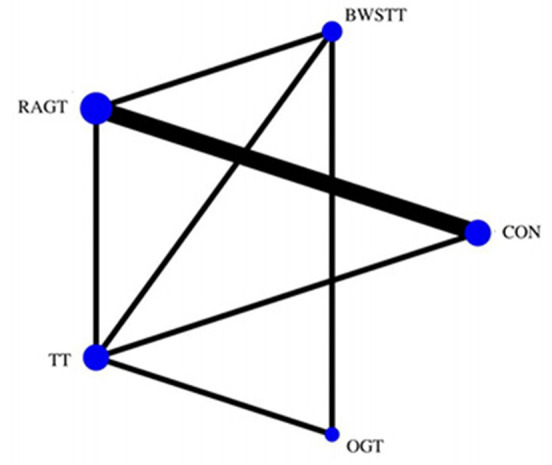
Evidence network of GMFM-E analysis. BWSTT, partial body weight supported treadmill training; C, conventional physical therapy; OGT, over ground gait training; RAGT, robot-assisted gait training; TT, treadmill training.

GMFM corresponding to dimensions D and E, were reported in nine studies involving five interventions, including CON, BWSTT, RAGT, TT, and OGT with a total of 243 patients. The results indicated that the largest number of studies comparing RAGT with CON was 3. Besides, the largest sample size of studies (107 cases) compared RAGT with CON, and the evidence networks are illustrated in Figures 4, 5, respectively. Five interventions were directly compared in a NMA of included studies. For dimension D, the results of the NMA showed no statistically significant difference with any two-way comparison between groups. For dimension E, the results of the NMA indicated that RAGT [MD = 10.45, 95% CI (2.51, 18.40), *P* < 0.05] was significantly more effective than CON, whereas the remaining groups did not show a difference with statistical significance (*P* > 0.05), as listed in Tables 3, 4.

**Table 3:**
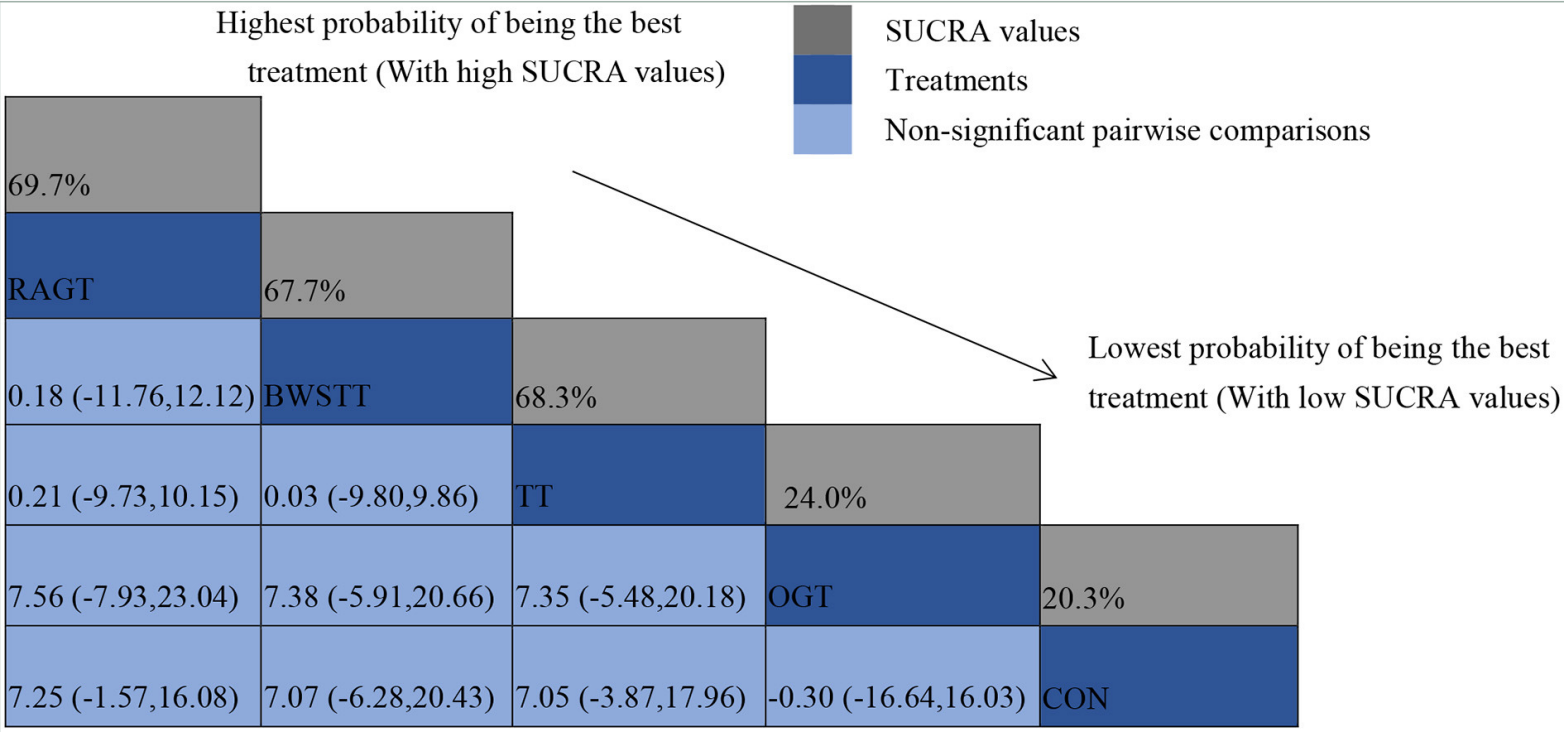
Relative effect sizes of efficacy at post-intervention according to network meta-analysis.

Interventions are orders in the rank of their chance of being the best intervention. Numbers in gray boxes are SUCRA (the surface under the cumulative ranking curve) values, which represented the rank of the treatment. BWSTT, partial body weight supported treadmill training; CON, conventional therapy; OGT, over ground gait training; RAGT, robot-assisted gait training; TT, treadmill training. Significant pairwise comparisons are highlighted in bold and underlined (MD with 95% CI).

**Table 4:**
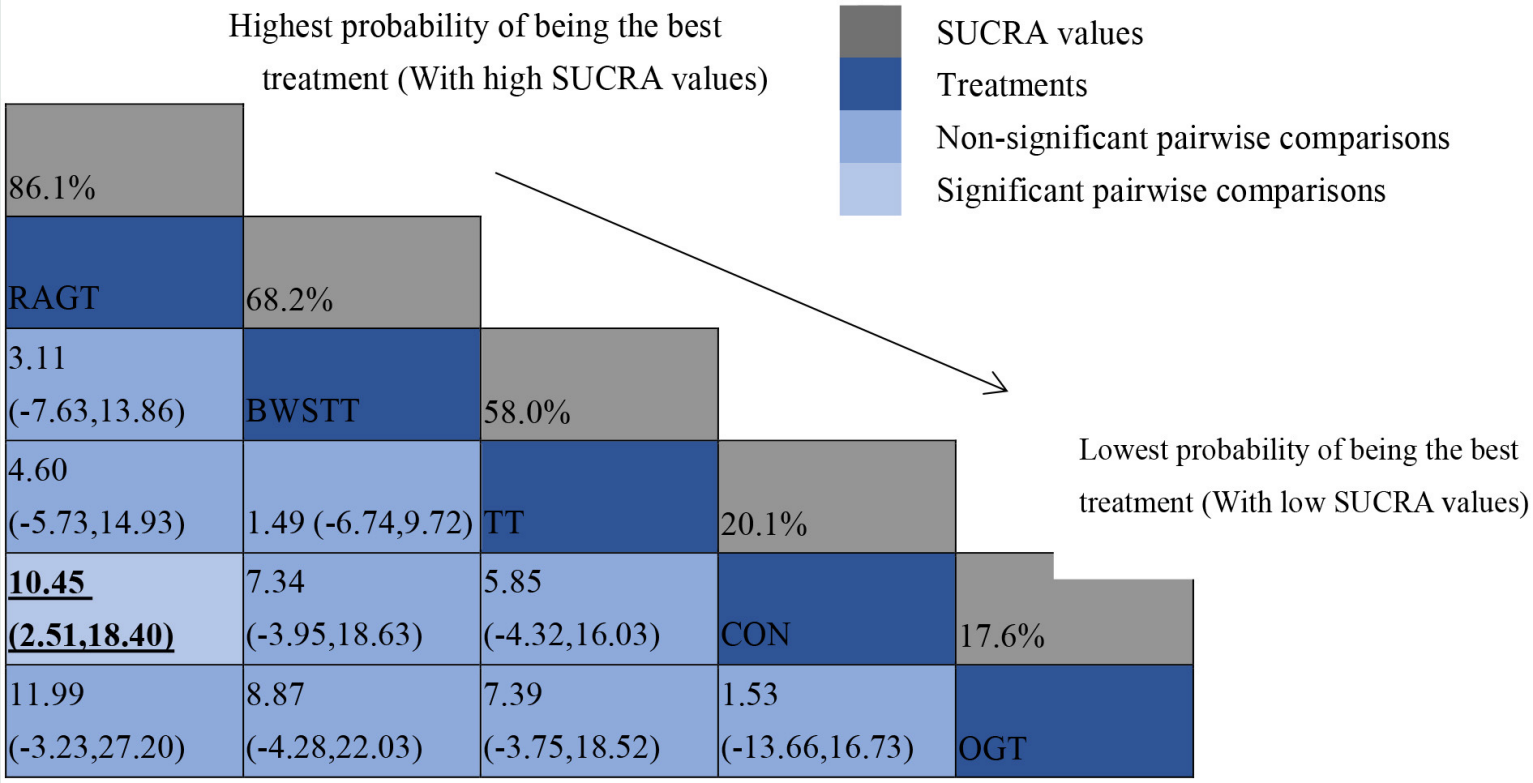
Relative effect sizes of efficacy at post-intervention according to network meta-analysis.

Interventions are orders in the rank of their chance of being the best intervention. Numbers in gray boxes are SUCRA (the surface under the cumulative ranking curve) values, which represented the rank of the treatment (MD with 95% CI). BWSTT, partial body weight supported treadmill training; C, conventional therapy; OGT, over ground gait training; RAGT, robot-assisted gait training; TT, treadmill training. Significant pairwise comparisons are highlighted in bold and underlined (MD with 95% CI).

All showed the highest likelihood of the optimal intervention for RAGT. The cumulative probability ranking was for dimension D: RAGT (69.7%) > TT (69.3%) > BWSTT (67.7%) > OGT (24%) > CON (20.3%); for dimension E: RAGT (86.1%) > BWSTT (68.2%) > TT (58%) > CON (20.1%) > OGT (17.6%), as presented in Supplementary Figures S5, S6. Comparison-corrected funnel plots (Supplementary Figures S7, S8) suggested a possible small sample effect or publication bias between studies. The results show that for the three closed loops involving outcome indicators for dimensions D and E, indicating that there is inconsistency in the loops and that the results need to be interpreted with caution, as detailed in Supplementary Figures S9, S10.

## Discussion

In this systematic review and NMA, 20 RCTs involving 516 CP patients were included. This is the first study to use the NMA to examine the relative effectiveness of different gait training on gait function in persons with CP. This study sought to summarize the available data to indicate that the greatest likelihood of being the best gait intervention to increase the gait velocity in children with CP lies in BWSTT (SUCRA = 91.7%). This study also confirmed that RAGT is most likely to improve GMFM in CP patients, in which the D and E dimensions of GMFM are included as they relate to motor function in gait and standing, for dimension D SUCRA (69.7%); for dimension E SUCRA (86.1%), the above very promising gait interventions show different effects depending on their individual characteristics or specific technique, which needs to be confirmed by further research with more robust evidence.

Due to the increasing number of interventions currently available to treat gait capacity in people with CP and the rapid growth in publications. There has been controversy as to whether different gait training is effective in increasing the gait velocity in CP patients, and best practice in rehabilitation requires adequate evidence. This study is required to consider which gait interventions are more appropriate for this particular population. Relevant to the main aim of this paper, sufficient evidence demonstrates that patients with CP improve their gait velocity and overall function with gait training, and previous meta-analyses have suggested that gait interventions outperform traditional rehabilitation in increasing the gait velocity of CP patients (11, 55). Although both studies have involved considerable articles and participants, they have not all included RCTs, and there is a risk of bias in the results. Our study further confirms that BWSTT may be the optimal intervention for increasing the gait velocity in patients with CP. BWSTT can reduce some of the patient's weight during gait training, reduce the load the patient has to overcome and ensure safety and stability during walking. Previously published systematic reviews and meta-analyses have shown that BWSTT is highly effective in increasing the gait velocity and other walking abilities in people with CP compared with conventional rehabilitation (15, 56, 57). A major hypothesis for the effectiveness of BWSTT in increasing the gait velocity in CP patients is that BWSTT gait interventions have a reduced double support phase time and a reduced reaction time compared with other gait interventions. BWSTT improves motor control by increasing the strength of the lower body muscles and the performance of the cardiorespiratory system more (58). The increased gait velocity in CP patients not only improves social participation and self-care, but also reduces the fear of falling (59–61).

In addition, this study found that the second most effective gait intervention to increase the gait velocity in children with CP was ECTT (SUCRA = 80.9%). Sensory feedback networks are generally impaired in people with CP, and increasing the number of external cues to exercise facilitates the learning and modification of desired movement patterns. Existing studies have suggested that external cueing exercises can be employed to restore neuroplasticity in damaged neurons and neural networks in CP patients (62). Visual or auditory cues can also provide a powerful signal for the reorganization of sensory-motor circuits in CP patients and help CP learn to establish Near-neurophysiological walking patterns (63). ECTT can provide a powerful motivation during walking in persons with CP, improve participants' attention and can come up better outcomes. However, the description of feedback methods was not always clear in the identified studies and to isolate any effects, the current study did not specifically further classify ECTT. The added value of innovations in ECTT is an emerging topic and can take on a great significance in pediatric rehabilitation.

For GMFM, this study suggests that RAGT is most likely to improve GMFM (latitude D and latitude E) in CP patients, evidence that was confirmed by a previous NMA (15). RAGT has a potential to improve gross function in persons with CP probably because the use of RAGT promotes physical and cognitive integration and provides a near-physiological gait pattern due to the intensive, repetitive, and task-oriented training it can offer (63). For the type of robot used in RAGT, most studies used “Lokomat” (38, 39, 42, 44, 53), one study used “3DCaLT” (47) and one study did not specify the specific type of robot applied (44). Some differences are found between the robots. There are some differences between the robots. The “Lokomat” consists of a suspended weight reduction support system, a lower limb exoskeleton gait corrector and a running platform and its control system (64). The “3DCaLT” is a custom-designed 3D cable-driven robotic gait training system (65). The scarcity of research evidence hinders the further classification of the types of robots used, thus limiting the proposed use of RAGT. Nevertheless, this study provides the latest insight into this gait intervention and may guide future primary research.

### Strengths and limitations

The greatest strength of this NMA is that it has been the first study to compare all major gait interventions for patients with CP. Based on the strict inclusion and exclusion criteria, we obtained a homogeneous sample including only RCTs to control for potential bias and to provide the best estimate of the impact of gait interventions on gait capacity in CP patients. Furthermore, gait interventions for people with CP are complex and multifaceted, and the very small number of relevant trials justifies the particular relevance of this NMA. This NMA includes only gait training interventions. By focusing on the above gait training interventions, we can differentiate between specific, various gait training methods, which can lay a basis for clinical practitioners prescribing therapeutic exercise. At the same time, the improved gait capacity of CP patients can improve their quality of life and reduce the burden on the patient's family and society. Despite the above advantages, there are some obvious limitations to our analysis. First, despite the good correlation loop between gait velocity and GMFM outcomes, the small number of reported GMFM studies and inconsistencies in the loop reveal that network inconsistencies may bias the above results. Therefore, we need to exercise caution when interpreting the above results. Second, although the NMA used all available data, due to the limited number of articles included, there was no specific description between RAGT and ECTT, and the evidence for indirect comparisons was not directly based on RCTs. As a result, it is recommended that future researchers include analysis and differentiation regarding external cues and their robotic devices in the analysis of valid rows for gait training. Third, we analysed the CON and OGT separately, because in our study CON stands for interventions such as static stretching of lower limbs' muscles and resistance training, etc. not include any form over ground gait training. It should be noted, however, that there are three articles (39, 42, 44) that do not clearly specify what conventional therapy consisted of. Fourth, we were unable to consider total duration of the intervention, duration and frequency of sessions and the intensity of exercises in our analysis since this information was lacking for some exercise modalities. Therefore, it is recommended that future researchers may need to be required to determine the duration and frequency of sessions and the intensity of exercise that are optimal for the promotion of gait ability in people with CP.

## Conclusion

Based on all findings together, this systematic review and NMA suggests that BWSTT may be the optimal intervention to increase the gait velocity of CP patients, while GMFM is most likely to be improved by RAGT. This study may provide strong evidence as to which gait intervention is the optimal intervention for improving walking ability in this special population and provide insight for subsequent research. Due to the quantitative and qualitative limitations of this study, randomized controlled trials with larger sample sizes, multiple centers, and high quality should be conducted to validate the above conclusion.

## Data availability statement

The original contributions presented in the study are included in the article/Supplementary material, further inquiries can be directed to the corresponding authors.

## Author contributions

GQ and XC served as principal authors, had full access to all the data in the study, take responsibility for the accuracy of the data analysis, contributed to the conception, design, draft of the manuscript, and the integrity of the data. KX, HT, and QM contributed to data acquisition and interpretation. GQ, ZO, and JL contributed to revise of the article and final approval. All authors contributed to the article and approved the submitted version.
