# Supplementary material for: Which gait training intervention can most effectively improve gait ability in patients with cerebral palsy? A systematic review and network meta-analysis
Source: Front Neurol. 2023 Jan 10;13:1005485. doi: 10.3389/fneur.2022.1005485 (PMC9871496; doi:10.3389/fneur.2022.1005485)
Supplement: Supplementary file 1 [file Data_Sheet_1.PDF]

Medline (via PubMed): 103

#6 #2 AND #3 AND #4 AND #5 103

#5"random*"[All Fields] AND "control*"[All Fields] Sort by: Most Recent 1,117,361

#4("walk*"[All Fields] AND ("aptitude"[MeSH Terms] OR "aptitude"[All Fields] OR "abilities"[All Fields] OR "ability"[All Fields])) OR ("gait*"[All Fields] AND ("aptitude"[MeSH Terms] OR "aptitude"[All Fields] OR "abilities"[All Fields] OR "ability"[All Fields])) 20916

#3 "gait*"[All Fields] OR (("treadmill"[All Fields] OR "treadmill s"[All Fields] OR "treadmills"[All Fields]) AND ("education"[MeSH Subheading] OR "education"[All Fields] OR "training"[All Fields] OR "education"[MeSH Terms] OR "train"[All Fields] OR "train s"[All Fields] OR "trained"[All Fields] OR "training s"[All Fields] OR "trainings"[All Fields] OR "trains"[All Fields])) OR "walk*"[All Fields] OR ("feedback"[MeSH Terms] OR "feedback"[All Fields] OR "feedbacks"[All Fields] OR "feedback s"[All Fields]) OR (("external"[All Fields] OR "externally"[All Fields] OR "externals"[All Fields]) AND ("cues"[MeSH Terms] OR "cues"[All Fields])) 492,027

#2 (((((((((((((((((((((((CP (Cerebral Palsy[All Fields])) OR (Dystonic-Rigid Cerebral Palsies[All Fields])) OR (Dystonic-Rigid Cerebral Palsy[All Fields])) OR (Mixed Cerebral Palsies[All Fields])) OR (Mixed Cerebral Palsy[All Fields])) OR (Monoplegic Infantile Cerebral Palsy[All Fields])) OR (Quadriplegic Infantile Cerebral Palsy[All Fields])) OR (Rolandic Type Cerebral Palsy[All Fields])) OR (Congenital Cerebral Palsy[All Fields])) OR (Little Disease[All Fields])) OR (Little's Disease[All Fields])) OR (Spastic Diplegia[All Fields])) OR (Monoplegic Cerebral Palsy[All Fields])) OR (Athetoid Cerebral Palsy[All Fields])) OR (Dyskinetic Cerebral Palsy[All Fields])) OR (Atonic Cerebral Palsy[All Fields])) OR (Hypotonic Cerebral Palsies[All Fields])) OR (Hypotonic Cerebral Palsy[All Fields])) OR (Diplegic Infantile Cerebral Palsy[All Fields])) OR (Spastic Cerebral Palsies[All Fields])) OR (Spastic Cerebral Palsy[All Fields])) OR (Monoplegic Cerebral Palsies[All Fields])) OR (Cerebral Palsy[All Fields])) 161,074

#1 "Cerebral Palsy"[MeSH]

Web of science: 400

#1 AND #2 AND #3 AND #4 400

#4 (TS=(random*)) AND TS=(control*) 1,432,731

#3(TS=(walk* ability)) OR TS=(gait* ability) 35,117

#2 (((((((((((((((((((((TS=(Cerebral Palsy)) OR TS=(Dystonic-Rigid Cerebral Palsies)) OR TS=(Dystonic-Rigid Cerebral Palsy)) OR TS=(Mixed Cerebral Palsies)) OR TS=(Mixed Cerebral Palsy)) OR TS=(Monoplegic Infantile Cerebral Palsy)) OR TS=(Quadriplegic Infantile Cerebral Palsy)) OR TS=(Rolandic Type Cerebral Palsy)) OR TS=(Congenital Cerebral Palsy)) OR TS=(Little Disease)) OR TS=(Little's Disease)) OR TS=(Spastic Diplegia)) OR TS=(Monoplegic Cerebral Palsy)) OR TS=(Athetoid Cerebral Palsy)) OR TS=(Dyskinetic Cerebral Palsy)) OR TS=(Atonic Cerebral Palsy)) OR TS=(Hypotonic Cerebral Palsies)) OR TS=(Hypotonic Cerebral Palsy)) OR TS=(Diplegic Infantile Cerebral Palsy)) OR TS=(Spastic Cerebral Palsies)) OR TS=(Spastic Cerebral Palsy)) OR TS=(Monoplegic Cerebral Palsies)) OR TS=(Cerebral Palsy) 2,094,270

#1 ((((TS=(gait*)) OR TS=(treadmill training)) OR TS=(walk*)) OR TS=(feedback)) OR TS=(external cues) 1,767,702

EMBASE: 167

#1 AND #2 AND #3 AND #4 167

#1 ‘gait*’ OR ‘treadmill training’ OR ‘walk*’ OR ‘feedback’ OR ‘external cues’ 772,834

#2 ‘Cerebral Palsy’ OR ‘CP’ 183,516

#3 ‘walk* ability’ OR ‘gait* ability’ 5,223

#4 ‘random*’ AND ‘control*’ 16,114,325

Cochrane: 233

#1 AND #2 AND #3 AND #4 235

#1 ((((((((((((((((((((((CP (Cerebral Palsy)) OR (Dystonic-Rigid Cerebral Palsies)) OR (Dystonic-Rigid Cerebral Palsy)) OR (Mixed Cerebral Palsies)) OR (Mixed Cerebral Palsy)) OR (Monoplegic Infantile Cerebral Palsy)) OR (Quadriplegic Infantile Cerebral Palsy)) OR (Rolandic Type Cerebral Palsy)) OR (Congenital Cerebral Palsy)) OR (Little Disease)) OR (Little's Disease)) OR (Spastic Diplegia)) OR (Monoplegic Cerebral Palsy)) OR (Athetoid Cerebral Palsy)) OR (Dyskinetic Cerebral Palsy)) OR (Atonic Cerebral Palsy)) OR (Hypotonic Cerebral Palsies)) OR (Hypotonic Cerebral Palsy)) OR (Diplegic Infantile Cerebral Palsy)) OR (Spastic Cerebral Palsies)) OR (Spastic Cerebral Palsy)) OR (Monoplegic Cerebral Palsies)) OR (Cerebral Palsy))

#2 (gait* OR treadmill training OR walk* OR feedback OR external cues)

#3 (walk* ability OR gait* ability)

#4 (random* AND control*)
