# Supplementary figures and images for: Which gait training intervention can most effectively improve gait ability in patients with cerebral palsy? A systematic review and network meta-analysis
Source: Front Neurol. 2023 Jan 10;13:1005485. doi: 10.3389/fneur.2022.1005485 (PMC9871496; doi:10.3389/fneur.2022.1005485)

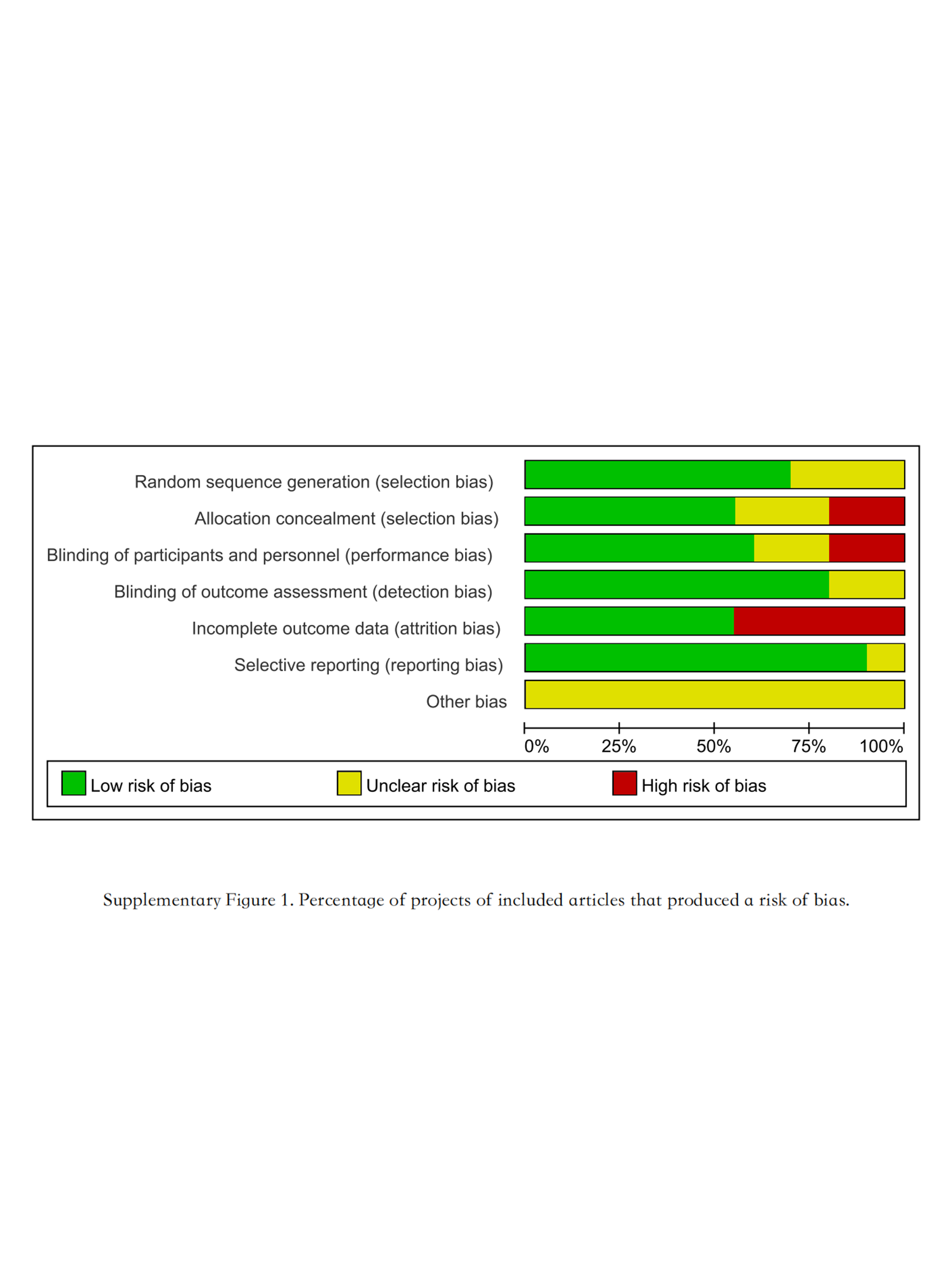

Supplement: Supplementary file 3 [file Image_1.TIF]

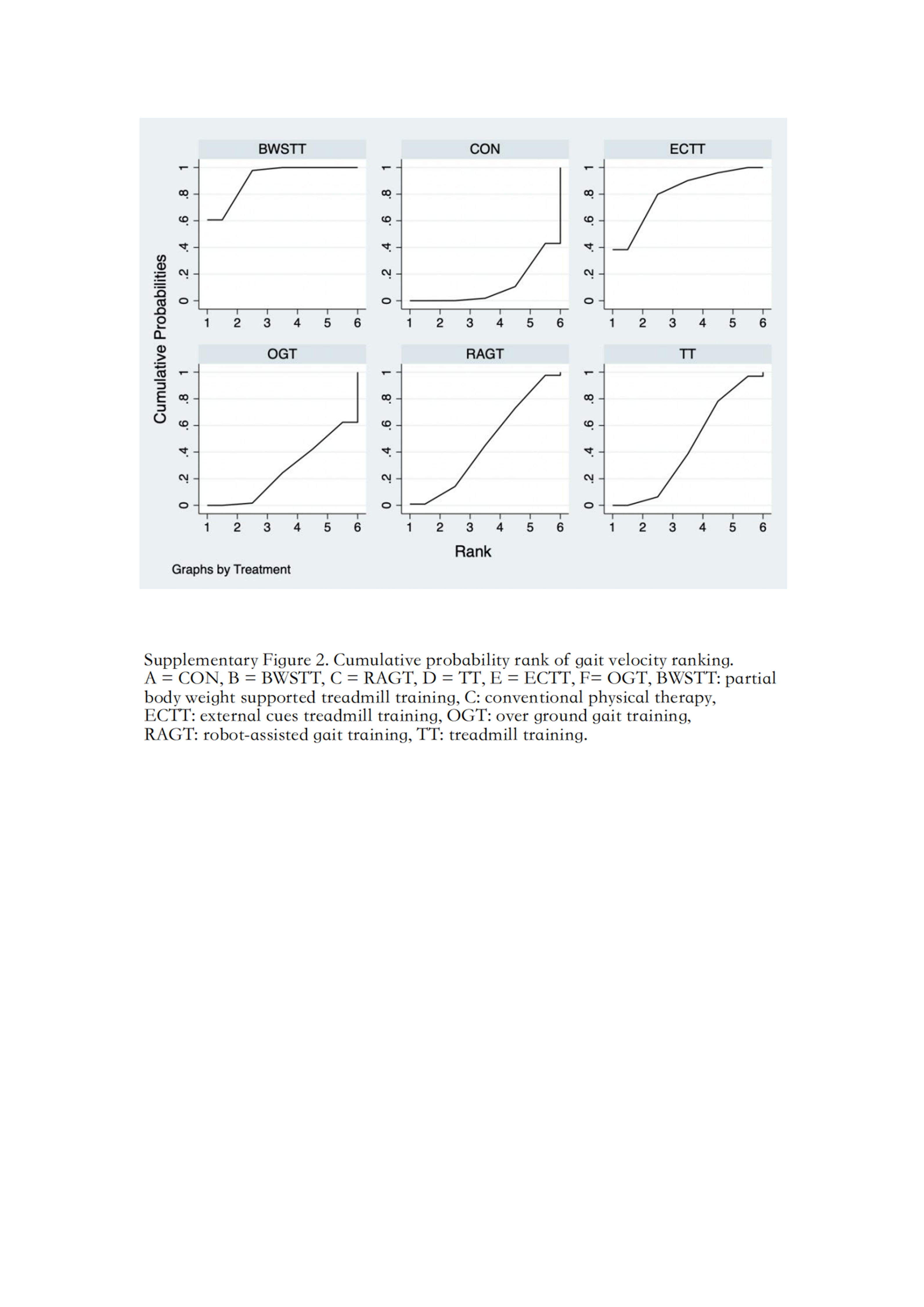

Supplement: Supplementary file 4 [file Image_2.TIF]

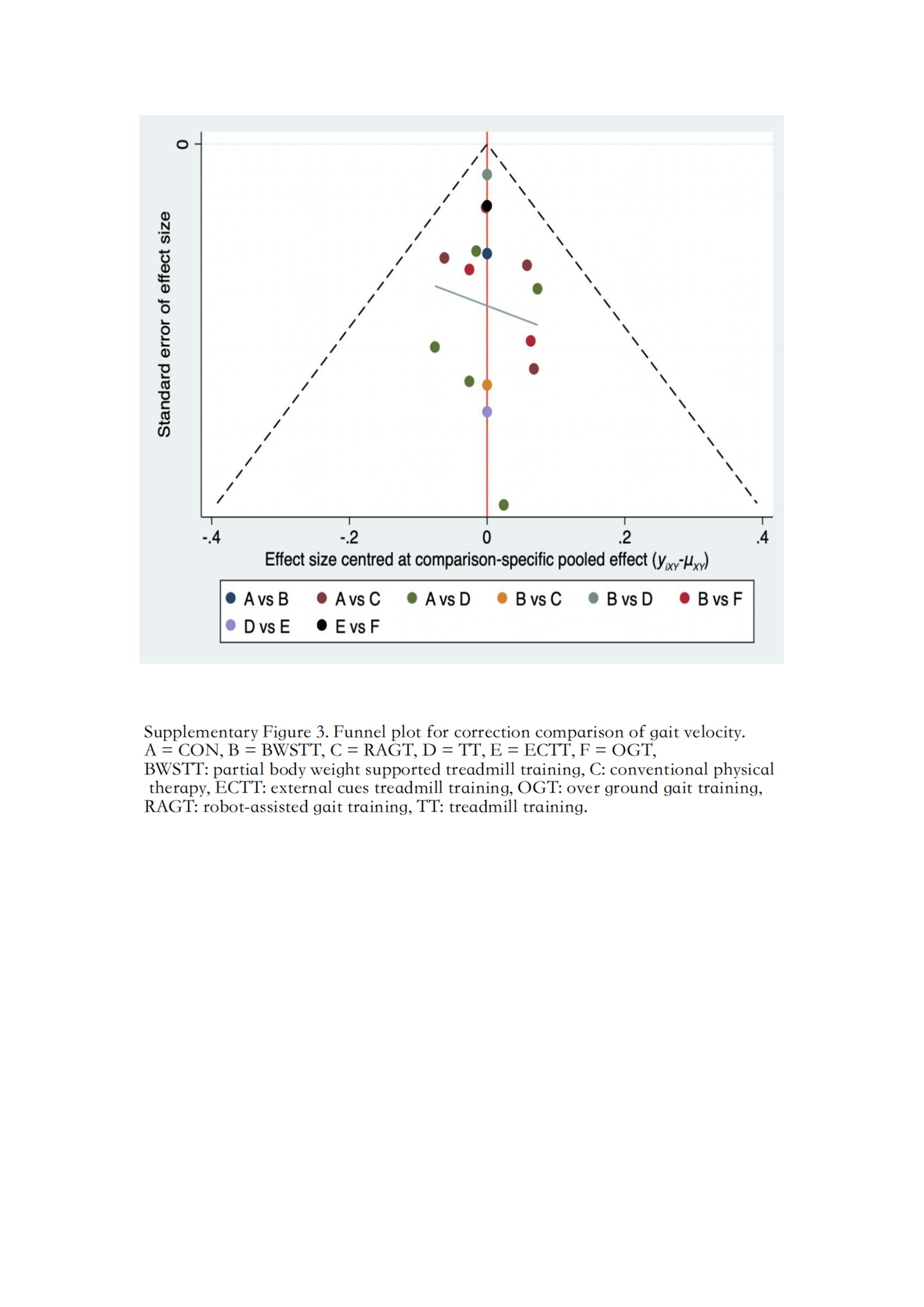

Supplement: Supplementary file 5 [file Image_3.TIF]

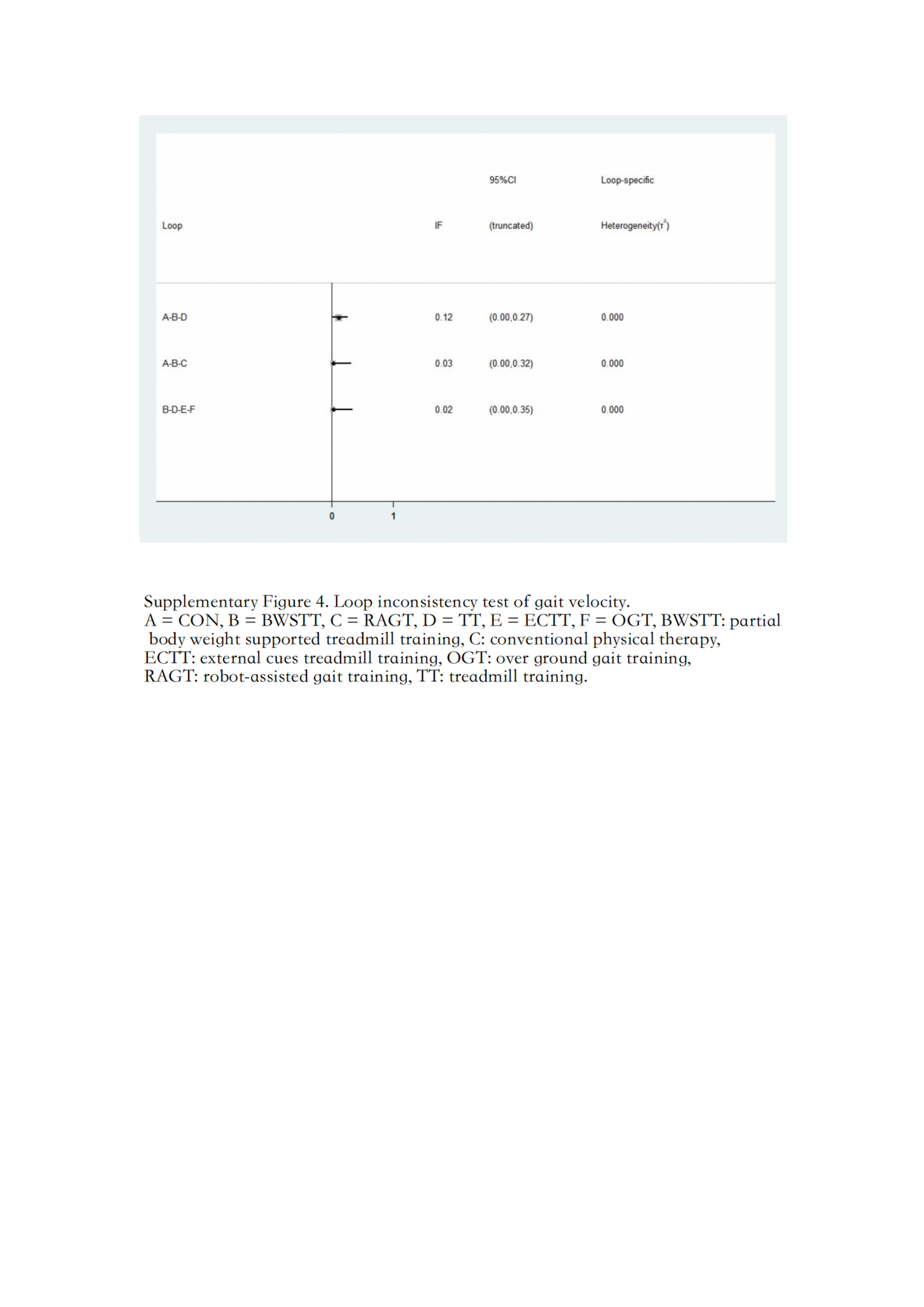

Supplement: Supplementary file 6 [file Image_4.TIF]

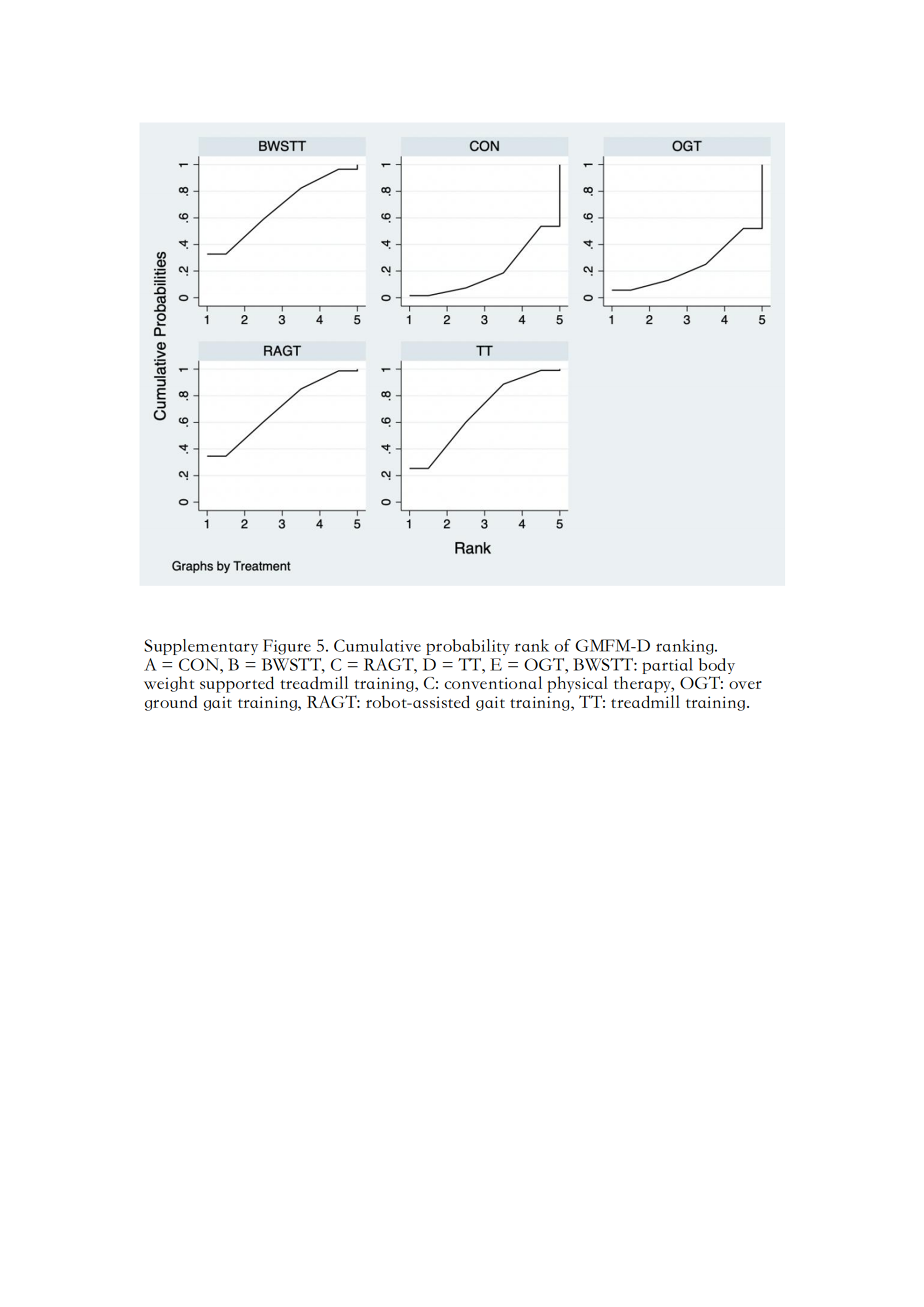

Supplement: Supplementary file 7 [file Image_5.TIF]

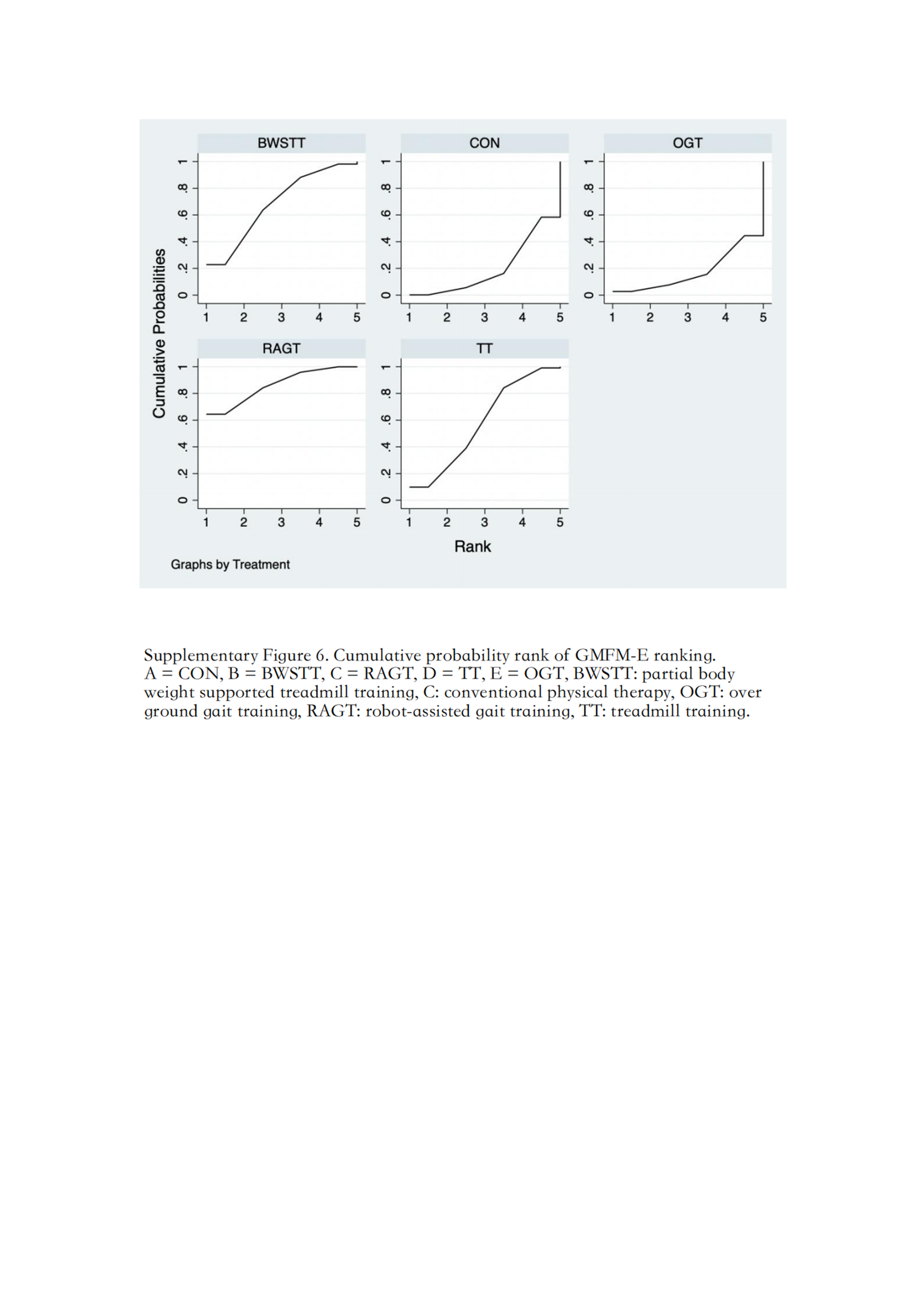

Supplement: Supplementary file 8 [file Image_6.TIF]

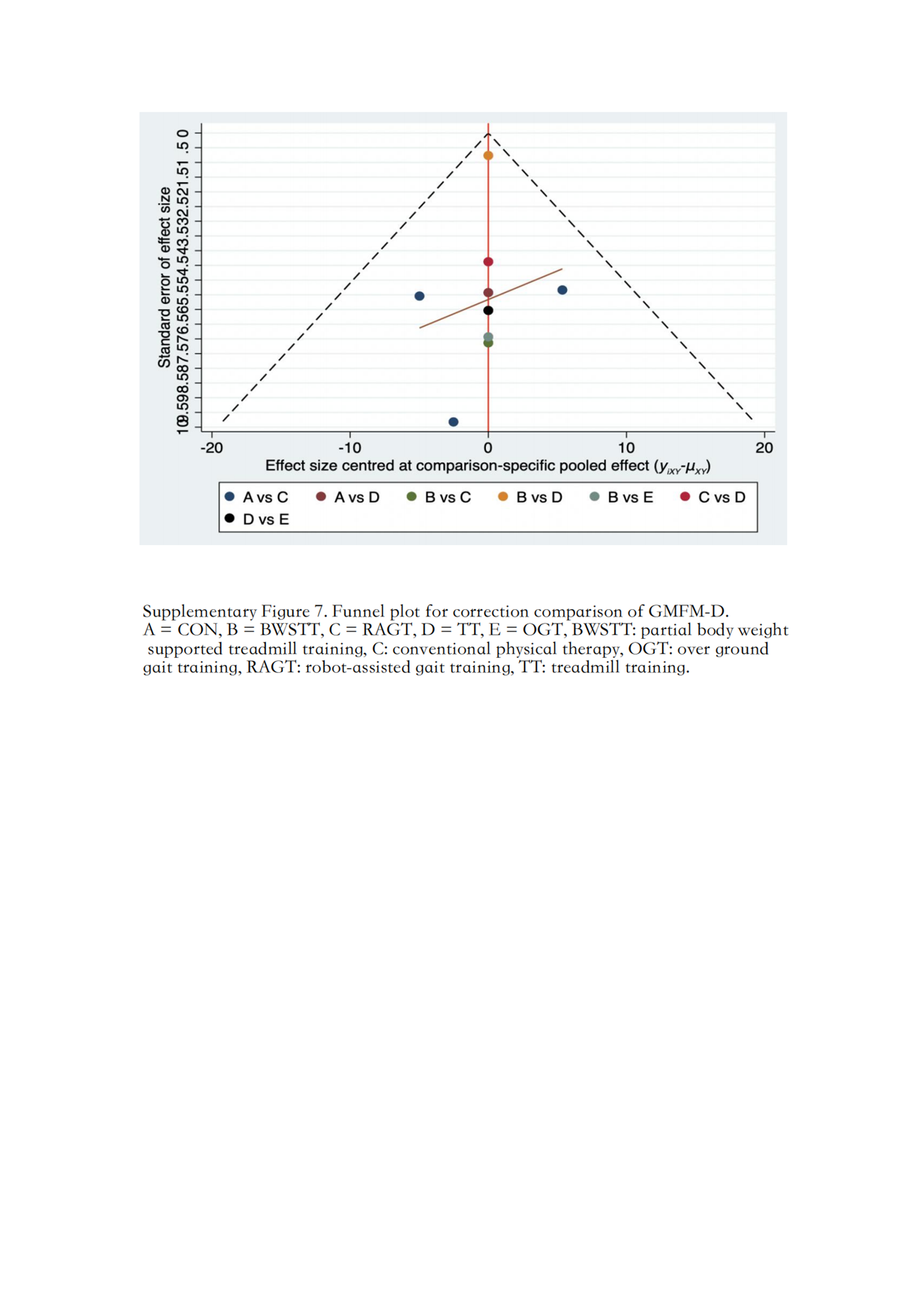

Supplement: Supplementary file 9 [file Image_7.TIF]

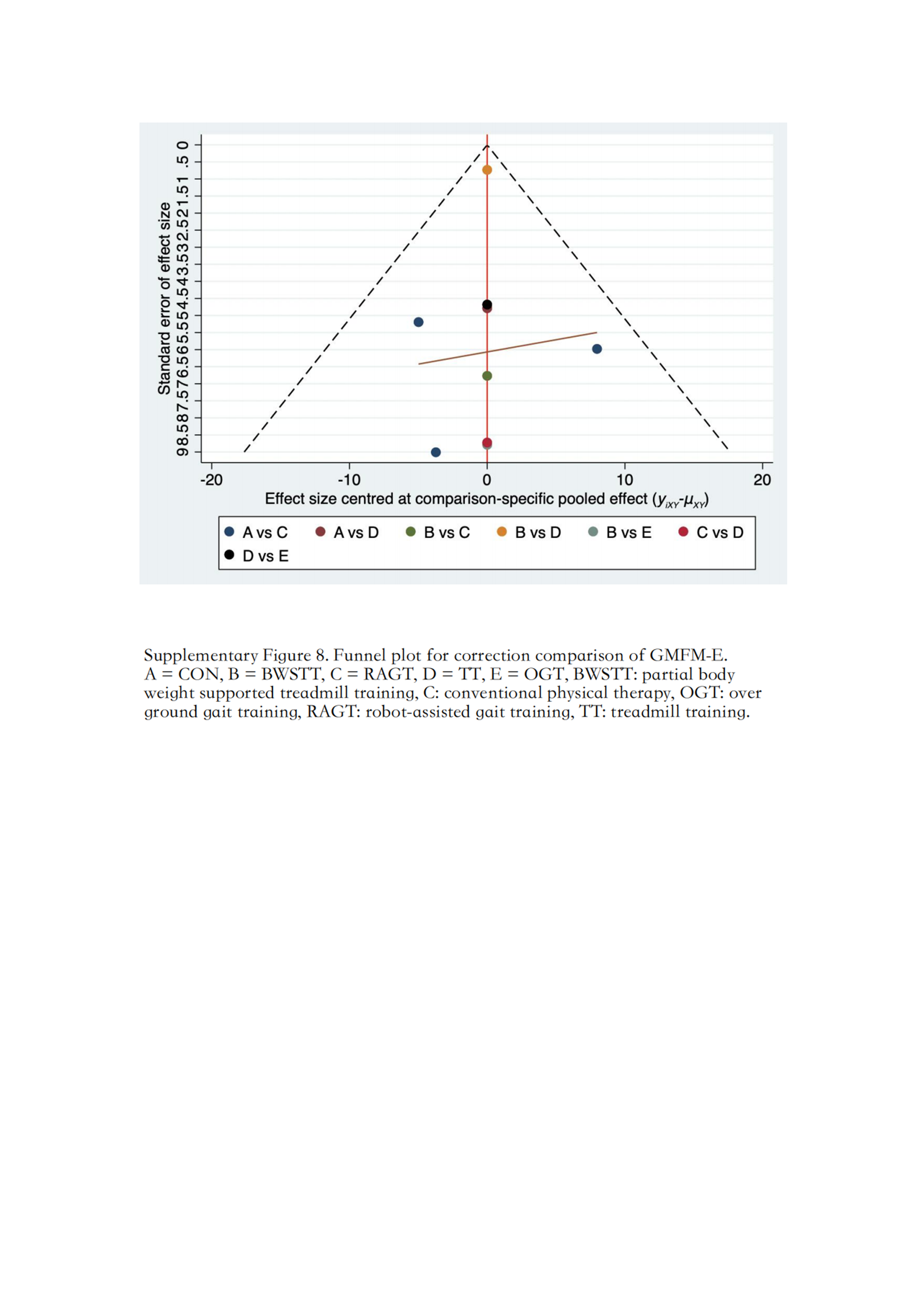

Supplement: Supplementary file 10 [file Image_8.TIF]

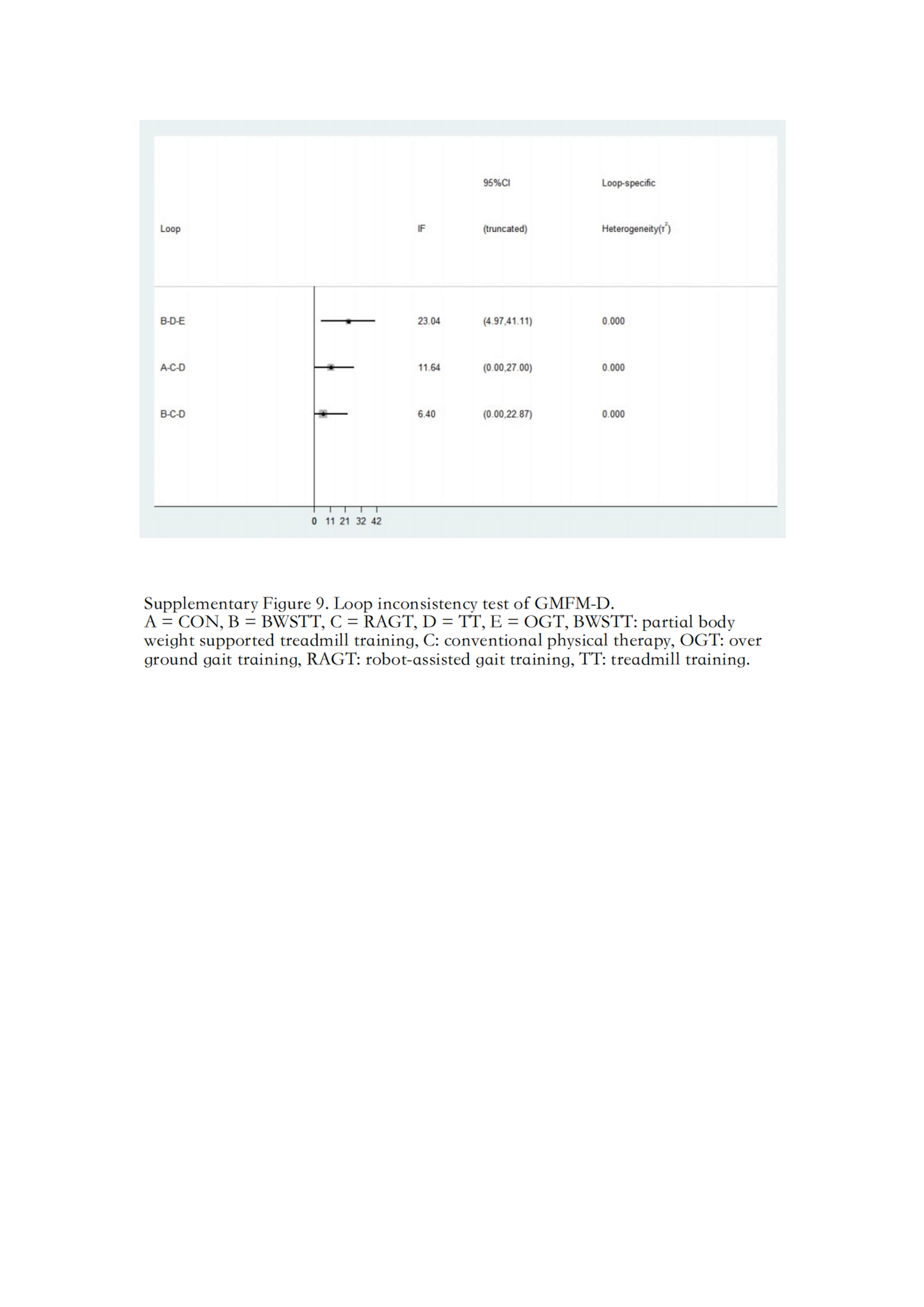

Supplement: Supplementary file 11 [file Image_9.TIF]

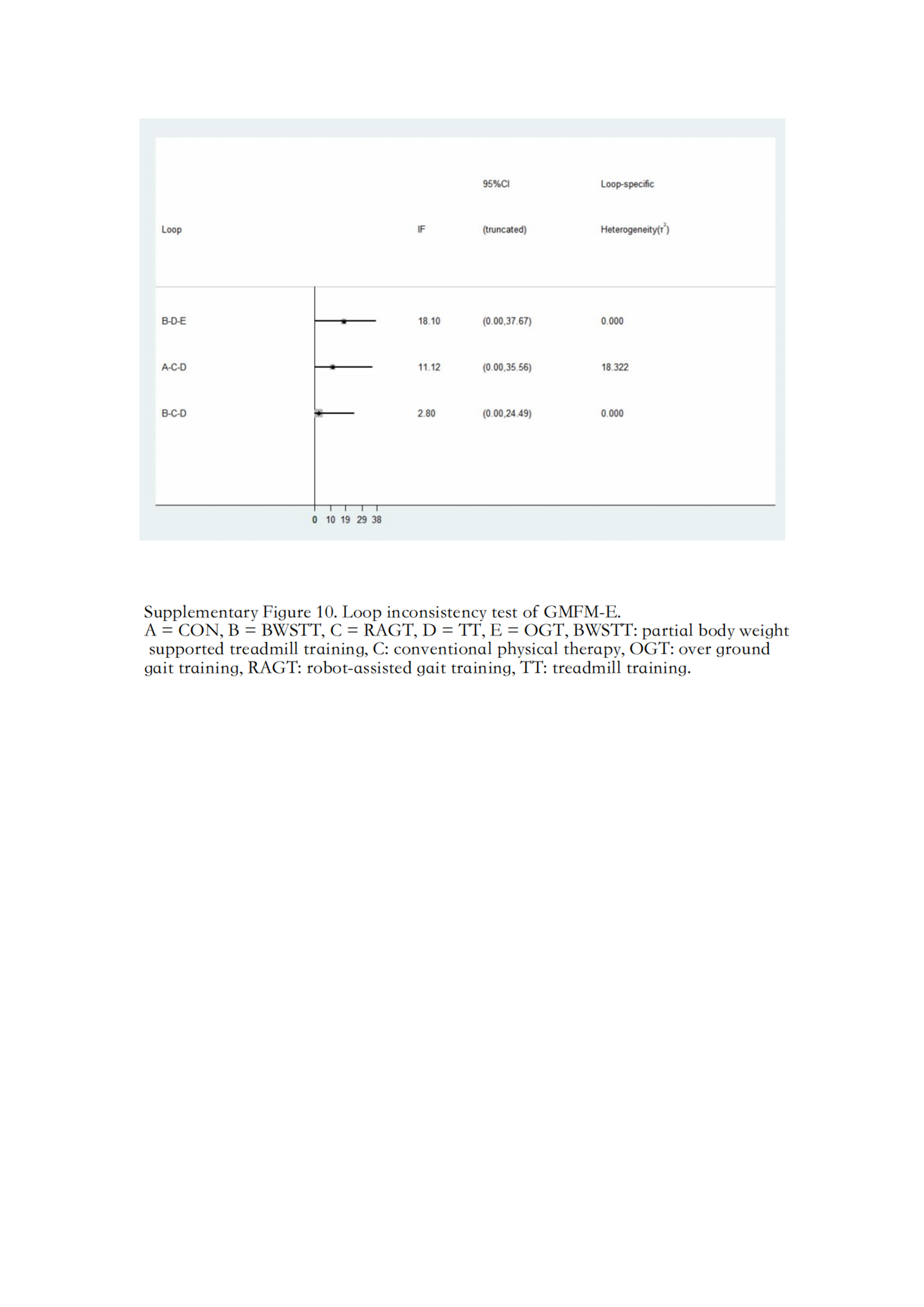

Supplement: Supplementary file 12 [file Image_10.TIF]
